# Angiotensin type 1a receptor-deficient mice develop diabetes-induced cardiac dysfunction, which is prevented by renin-angiotensin system inhibitors

**DOI:** 10.1186/1475-2840-12-169

**Published:** 2013-11-12

**Authors:** Qian Chen Yong, Candice M Thomas, Rachid Seqqat, Niketa Chandel, Kenneth M Baker, Rajesh Kumar

**Affiliations:** 1 Division of Molecular Cardiology, Department of Medicine, Texas A&M Health Science Center, College of Medicine; Scott & White; Central Texas Veterans Health Care System, 1901 South First Street, Building 205, Temple, Texas 76504, USA; 2 Current address: SENESCYT/Proyecto Prometeo, Laboratorio de Biotecnología Humana, Escuela Politécnica del Ejército, Sangolquí, Ecuador

**Keywords:** Renin-angiotensin system, Intracrine, Renin inhibitor, Diabetic cardiomyopathy kallikrein, Kininogen, Kinin B2 receptor

## Abstract

**Background:**

Diabetes-induced organ damage is significantly associated with the activation of the renin-angiotensin system (RAS). Recently, several studies have demonstrated a change in the RAS from an extracellular to an intracellular system, in several cell types, in response to high ambient glucose levels. In cardiac myocytes, intracellular angiotensin (ANG) II synthesis and actions are ACE and AT_1_ independent, respectively. However, a role of this system in diabetes-induced organ damage is not clear.

**Methods:**

To determine a role of the intracellular ANG II in diabetic cardiomyopathy, we induced diabetes using streptozotocin in AT_1a_ receptor deficient (AT_1a_-KO) mice to exclude any effects of extracellular ANG II. Further, diabetic animals were treated with a renin inhibitor aliskiren, an ACE inhibitor benazeprilat, and an AT_1_ receptor blocker valsartan.

**Results:**

AT_1a_-KO mice developed significant diastolic and systolic dysfunction following 10 wks of diabetes, as determined by echocardiography. All three drugs prevented the development of cardiac dysfunction in these animals, without affecting blood pressure or glucose levels. A significant down regulation of components of the kallikrein-kinin system (KKS) was observed in diabetic animals, which was largely prevented by benazeprilat and valsartan, while aliskiren normalized kininogen expression.

**Conclusions:**

These data indicated that the AT_1a_ receptor, thus extracellular ANG II, are not required for the development of diabetic cardiomyopathy. The KKS might contribute to the beneficial effects of benazeprilat and valsartan in diabetic cardiomyopathy. A role of intracellular ANG II is suggested by the inhibitory effects of aliskiren, which needs confirmation in future studies.

## Background

Diabetic cardiomyopathy, defined as ventricular dysfunction that occurs independent of vascular or valvular pathology, is a major morbidity factor in diabetic patients [1,2]. Several mechanisms have been implicated in the development of diabetic cardiomyopathy, including upregulated oxidative stress, impaired calcium homeostasis, and activation of the renin-angiotensin system (RAS) [3]. Clinical and experimental studies have shown beneficial effects of RAS inhibitors in diabetes-induced organ damage [4]. Currently, two classes of RAS inhibitors, angiotensin type 1 receptor (AT_1_) blockers (ARBs) and ACE inhibitors, are used in the clinic. However, cardiovascular morbidity and mortality remain higher in diabetic patients on RAS inhibitors compared to non-diabetics [5]. The latter observations have suggested the existence of residual risk in patients, which might be related to insufficient RAS blockade, among other factors. With regard to insufficient RAS blockade by ACE inhibitors and ARBs, we described an intracellular cardiac RAS that is significantly upregulated in diabetes [6-9]. An intracrine role of ANG II has also been described by other investigators [10,11]. The intracellular RAS in cardiac myocytes is not inhibited by ACE inhibitors and ARBs, due to chymase-mediated synthesis and AT_1_–independent actions of intracellular ANG II. However, a renin inhibitor prevents both intra- and extra-cellular ANG II synthesis. We reported that a renin inhibitor was more effective than an ACE inhibitor or ARB in preventing diabetes-induced cardiac superoxide production, apoptosis and fibrosis [8], suggesting the intracellular RAS as a possible residual risk factor in diabetes. To determine whether the intracellular cardiac RAS has a role in diabetes-induced cardiac dysfunction, we induced diabetes in AT_1a_ receptor-deficient (AT_1a_-KO) mice by streptozotocin (STZ). Our hypothesis was that in the absence of extracellular ANG II-mediated effects in these animals, development of cardiac dysfunction and prevention of the latter by a renin inhibitor would indicate involvement of intracellular ANG II in diabetic cardiomyopathy. We report that AT_1a_-KO mice develop diabetes-induced cardiac dysfunction, which is prevented by a renin inhibitor, as well as by an ACE inhibitor and ARB.

## Materials and methods

### Animals

**Figure 1:**
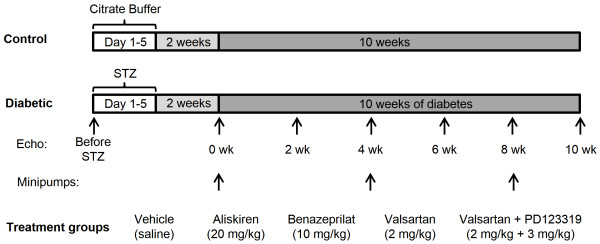
**Study design.** Twelve wk old male AT_1a_ receptor knockout (AT_1a_-KO) mice were injected with either 0.1 M citrate buffer (pH 4.5) or streptozotocin (50 mg/kg) for five days. After 2 wks, animals with blood glucose levels of ≥ 250 mg/dl were considered diabetic. Echocardiographic measurements were taken in all groups at the beginning (Before STZ), onset of diabetes (0 wk), and at two wk intervals thereafter. Osmotic minipumps containing one of the drugs were implanted subcutaneously at 0 wk and replaced after 4 and 8 wks. At the conclusion of the study (10 wks), blood pressure was measured by carotid artery cannulation and tissues were collected.

All animal protocols were approved by the Institutional Animal Care and Use Committee and conformed to the National Institutes of Health guidelines. Male AT_1a_-KO mice were purchased from the Jackson Laboratory (stock number 002682, Bar Harbor, Maine) and fed *ad libitum*. At 12 wks of age, animals were randomized into control and diabetic groups (Figure 1). Animals in the diabetic group received STZ (50 mg kg^-1^ day^-1^; zanosar) intraperitoneally (i.p.) for 5 consecutive days, while those in the control group received 0.1 M sodium citrate buffer (pH 4.5). After 2 wks, mice with a blood glucose value of ≥ 250 mg/dl were considered diabetic. The diabetic group was divided into 5 subgroups (n = 10) that were treated with either vehicle or RAS inhibitors, as follows: 1) STZ + saline (STZ + Veh), 2) STZ + Aliskiren (20 mg/kg, STZ + Alsk), 3) STZ + Benazeprilat (10 mg/kg, STZ + Benz), 4) STZ + Valsartan (2 mg/kg, STZ + Vals), and STZ + PD123319 (3 mg/kg) + Valsartan (2 mg/kg, STZ + PD + Vals). The drug dosages were based on our previously published study [12]. Drugs were delivered by subcutaneous osmotic minipumps (ALZET 1004, 0.11 μl/hr), for 10 wks, with minipump replacement every 4 wks. Echocardiography was performed at the beginning of diabetes (0 wk) and every 2 wks thereafter. At the end of the study (10 wks of diabetes), the right carotid artery was cannulated and arterial pressure measured under isoflurane/oxygen (1.5/2%) anesthesia with a blood pressure analyzer (BPA 400, Micro-Med, Louisville, KY). Studies have shown a close correlation between systolic blood pressure measured under the conscious state and steady isoflurane anesthesia and that the latter can be reliably used to monitor changes in blood pressure in mice [13]. Hearts were immediately collected and processed for either cardiac myocytes isolation or fixed with paraformaldehyde for staining. In addition to the 10 wks study, a short study of one wk was performed to measure cardiac RAS activation.

### Echocardiographic measurements

Transthoracic echocardiography was performed using a VisualSonics Vevo 2100 with a 35-MHz probe. Briefly, mice were anesthetized with 3-5% isoflurane (with 2% oxygen) that was reduced to 1.5% to maintain the heart rate at about 450 beats per minute. The heart was imaged in the 2-dimensional, short-axis, and 4-chamber view. Left ventricular (LV) fractional shortening (FS), ejection fraction (EF), stroke volume (SV), cardiac output (CO), LV internal dimension at end-diastole (LVIDd), LV internal dimension at end-systole (LVIDs), LV posterior wall thickness at end-diastole (LVPWd), LV posterior wall thickness at end-systole (LVPWs), interventricular septum thickness at end-diastole (IVSd), intra-ventricular septum thickness at end-systole (IVSs), isovolumic relaxation time (IVRT), isovolumic contraction time (IVCT), early diastolic (E’) and late diastolic (A’) mitral annulus tissue Doppler velocities, and peak velocity of early (E) and late (A) LV filling waves were measured.

### Isolation of adult mouse cardiomyocytes

Adult mouse cardiomyocytes were isolated using a temperature-controlled (37°C) Langendorff’s perfusion system, as previously described with some modifications [14]. Briefly, hearts were perfused with perfusion buffer for 5 min, followed by digestion with collagenase II (0.17% W/V, 330 U/mg, Worthington Biochemical Corp), for 10 min, at a rate of 3 ml/min. After stopping digestion with a serum- and calcium-containing buffer, cells were washed with PBS and collected by centrifugation (180 rcf, 1 min). One portion of cardiac myocytes was snap frozen in liquid nitrogen for protein analysis or ANG II ELISA measurement, and the other was stored in RNAlater solution for RNA analysis.

### Real-time PCR

Gene expression of angiotensinogen (AGT), renin, AT_1a_, AT_1b_, tissue kallikrein, kininogen 2, prorenin receptor (PRR), ACE2, and Mas was determined using TaqMan assays (Applied Biosystems). RNA was extracted using an RNeasy Fibrous Tissue Kit (Qiagen). cDNA was made using a High Capacity cDNA Reverse Transcription Kit (Applied Biosystems). Real-time PCR was performed in 20 μl reaction containing cDNA, TaqMan Universal PCR master mix, and 20X specific gene expression assay mix (Applied Biosystems). Data were normalized to 18S mRNA. Each sample was run in duplicate, and the threshold cycle, ΔCt, was calculated as Ct (target gene) - Ct (18S). The relative changes in target gene in different treatment groups were determined by the formula 2^-ΔΔ Ct^, where ΔΔ Ct = ΔCt (control) - ΔCt (treatment group). The efficiency of AT_1a_ and AT_1b_ PCR was determined using a 10-fold dilution series of mouse adrenal gland cDNA. The slope of the standard curve was determined and the efficiency calculated using the formula E = 10 ^(−1/slope)^ -1.

### Western blot analysis

Protein expression of renin, ANG II Type 2 receptor (AT_2_), and bradykinin Type 2 (B2) receptor was assessed with the standard Western immunoblotting technique. Briefly, cardiac myocytes were sonicated in ice-cold lysis buffer (Cell Signaling) supplemented with protease and phosphatase inhibitor cocktails (Roche Applied Science). Homogenates were centrifuged at 16,000 g and protein concentration in the supernatant was determined using DC™ protein assay kit (BioRad). Equal amounts of cell lysate (60 μg) or plasma (0.05 μl) were separated on 4-20% SDS-polyacrylamide gels (120 V, 1 hr) and transferred to nitrocellulose membranes (27 V, 2 hrs). Blots were probed with anti-renin (Sigma-Aldrich), anti-AT_2_, and anti-B2 receptor (Santa Cruz Biotechnology) antibodies. Equal protein loading was confirmed by Ponceau staining, because GAPDH and beta-actin levels in the hearts of AT_1a_-KO mice were significantly affected by diabetes or the treatments (data not shown). Protein bands were detected using secondary antibodies labeled with infrared dye 680/800 and the enhanced Odyssey Infrared Imaging System (LI-COR, Biosciences).

### ANG II measurement

ANG II levels in isolated cardiac myocytes were measured by a competitive quantitative ELISA, following extraction with 1 M acetic acid and purification over a reverse phase C18 column, as described previously [15]. Since the cardiac myocyte isolation procedure involves perfusion of the heart, enzymatic digestion, and washing of the dispersed myocytes with PBS, the steps would wash-off extracellular ANG II; ANG II measured this way represents intracellular ANG II. The above intracellular ANG II method had previously been validated by mass spectrometry and confocal microscopy [9]. ANG II data have been presented in terms of per unit heart weight to be consistent with the literature.

### Reactive oxygen species staining

Hearts were fixed in 4% paraformaldehyde and frozen in O.C.T. compound (Tissue-Tek). Frozen sections (20 μm) were incubated with 10 μM dihydroethidium (DHE, Sigma-Aldrich), at 37°C, for 30 min in a humidified chamber protected from light. Fluorescent images (60X) were obtained with a Leica TCS SP5X confocal microscope and analyzed using ImageJ. Mean DHE fluorescence was calculated by subtracting integrated density of the background signal from the integrated density of the fluorescent staining for 10 fields/heart, 5 hearts/group, and normalized to control.

### Apoptosis

Hearts were fixed in 10% paraformaldehyde and paraffin embedded. Paraffin embedded sections (5 μm) were incubated at 60°C for 15 minutes, de-waxed, and rehydrated. Apoptosis was detected using terminal deoxynucleotide transferase-mediated dUTP nick-end labeling (TUNEL) assay, according to the manufacturer’s instructions (In situ Cell Death Detection Kit, Roche). Actin filaments and nuclei of cardiac myocytes were counterstained using Alexa Fluor 546 phalloidin (Invitrogen) and DAPI, respectively. Positively stained nuclei were counted from 6–12 sections per heart and 3 hearts per treatment group.

### Statistical analysis

All data were expressed as the mean ± SEM. One-way ANOVA with Tukey’s post hoc test or multiple comparisons using two-way ANOVA with the Bonferroni post hoc test, where appropriate, were used for statistical analysis (GraphPad). P <0.05 was considered statistically significant.

## Results

### Diabetes activates the cardiac intracellular RAS

**Figure 2:**
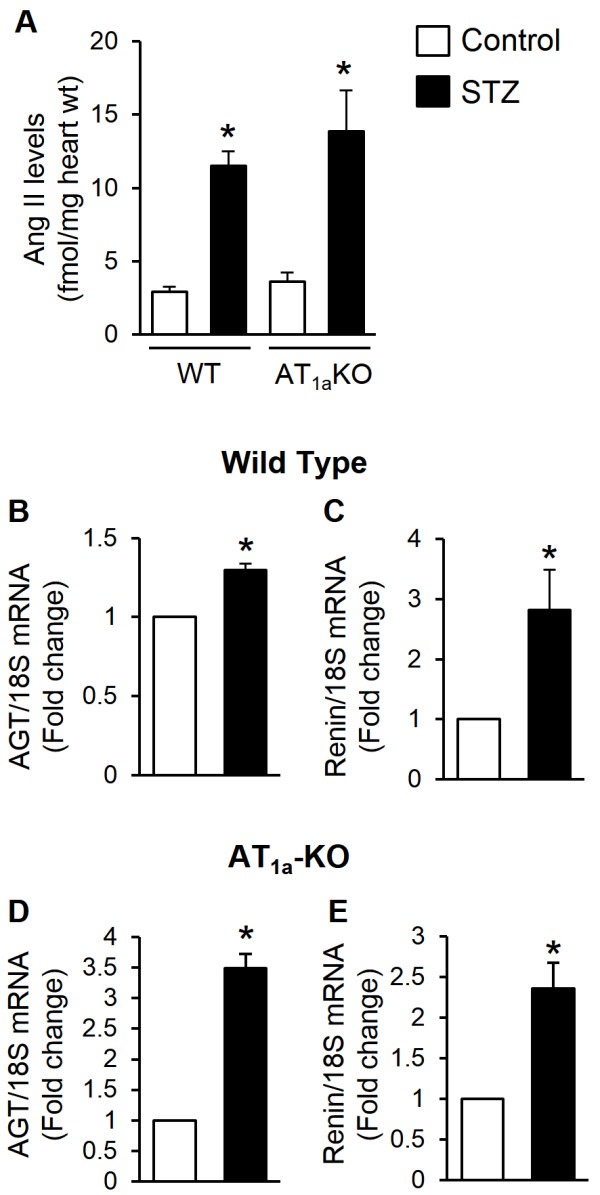
**Activation of the renin-angiotensin system.** Activity of the RAS was measured in cardiac myocytes isolated from wild type **(A-C)** and AT_1a_-KO **(A, D-E)** mice after 1 wk of diabetes. ANG II **(A)** was extracted from cardiac myocytes and measured using a competitive ELISA. Angiotensinogen (AGT, **B** and **D**) and renin **(C and E)** expression was measured in control and diabetic (STZ) samples, by real-time RT-PCR and normalized to 18S mRNA. Values are expressed as the fold change compared to control ± SEM. *P < 0.05 vs. control.

We had previously demonstrated intracellular ANG II synthesis in neonatal and adult cardiac myocytes following stimulation with high glucose in vitro and in rat hearts after one wk of diabetes [8,9]. In these studies, an ARB, candesartan, was used to prevent uptake of extracellular ANG II, to distinguish intracellularly synthesized ANG II from extracellular ANG II internalized by cells. To further confirm intracellular synthesis of ANG II and activation of the RAS, we produced diabetes in AT_1a_-KO and WT mice for one wk and measured ANG II levels in cardiac myocytes isolated from control and diabetic animals. As shown in Figure 2, a several fold increase in intracellular ANG II levels was observed in both AT_1a_-KO and WT diabetic mice, compared to non-diabetic controls. Additionally, AGT and renin gene expression was increased in cardiac myocytes of diabetic animals compared to controls. These results demonstrated activation of the cardiac intracellular RAS by hyperglycemia in AT_1a_-KO mice.

### Diabetes induces diastolic dysfunction in AT_1a_-KO mice, which is prevented by aliskiren, benazeprilat, and valsartan

**Figure 3:**
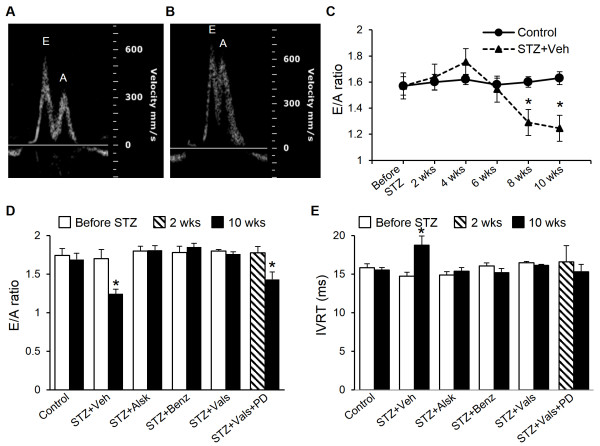
**Measurement of diastolic function by echocardiography.** Representative pulsed-wave Doppler images of Mitral valve flow of Control and STZ + Veh mice at 10 wks of diabetes (**A** and **B**, respectively). Temporal recordings of Mitral valve flow velocity (E/A) in control and diabetic mice implanted with saline minipumps (STZ + Veh), demonstrating development of diastolic dysfunction **(C)**. Mitral valve flow velocity **(D)** and isovolumic relaxation time **(E)** of Control, STZ + Veh, and those treated with aliskiren (STZ + Alsk), benazeprilat (STZ + Benz), valsartan (STZ + Vals), and valsartan + PD123319 (STZ + Vals + PD); before STZ treatment and at 10 wks after becoming diabetic. 2 wk values for STZ + Vals + PD are shown, as we could not collect reliable (before STZ) data in this group. Values are expressed as the mean ± SEM. *P < 0.05 vs. Control **(C)** or respective Before STZ **(D and E)**.

Others and we have shown prevention of cardiac dysfunction by ARBs and ACE inhibitors in animal models of diabetes [12]. To determine whether AT_1a_-KO mice would develop diastolic dysfunction, we used echocardiography to monitor ventricular function of control and diabetic mice for up to 10 wks following the onset of diabetes. Further, we treated diabetic mice with a renin inhibitor (aliskiren), ACE inhibitor (benazeprilat), and ARB (valsartan) to determine whether these agents would have a protective effect in the absence of AT_1a_ receptor. Diastolic function was evaluated by measuring the ratio of mitral valve flow velocities (E/A ratio) and isovolumic relaxation time (IVRT). As shown in Figure 3C and D, the E/A ratio was significantly lower at 8 and 10 wks of diabetes, compared to before STZ. Further, IVRT increased significantly after 10 wks of diabetes (Figure 3E, only 10 wk data shown). Intriguingly, treatment with all three classes of RAS inhibitors, including an ARB, completely prevented the progression of diastolic dysfunction. Blocking AT_2_ receptor with PD123319 in the presence of valsartan reduced the protective effects of the latter on E/A (Figure 3D), suggesting a beneficial role of AT_2_.

### Diabetes induces systolic dysfunction in AT_1a_-KO mice, which is prevented by aliskiren, benazeprilat, and valsartan

**Figure 4:**
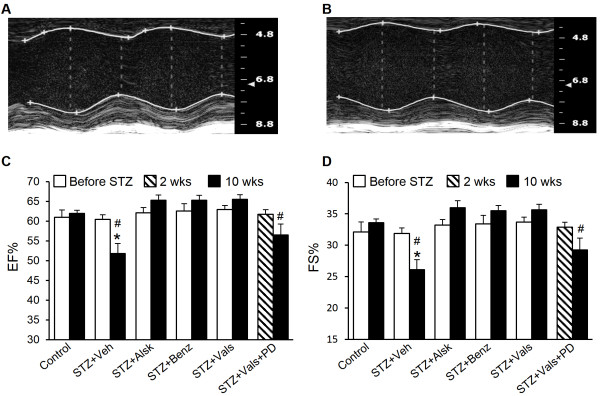
**Measurement of systolic function by echocardiography.** Representative M-mode short axis views of Control and STZ + Veh mice at 10 wks of diabetes (**A** and **B**, respectively). Ejection fraction **(C)** and fractional shortening **(D)** of Control, STZ + Veh, STZ + Alsk, STZ + Benz, STZ + Vals, and STZ + Vals + PD, before STZ treatment and at 10 wks after becoming diabetic. 2 wk values for STZ + Vals + PD are shown, as we could not obtain reliable (before STZ) data in this group. Values are expressed as the mean ± SEM. *P < 0.05 vs. respective Before STZ. ^#^*P* < 0.05 vs. Control (10wk).

In addition to diastolic dysfunction, we observed impairment of systolic function in diabetic AT_1a_-KO mice, as determined by measurement of EF and FS (Figure 4). Similar to WT animals [12], systolic dysfunction in the diabetic AT_1a_-KO animals manifested at 10 wks after the establishment of diabetes. All three RAS inhibitors protected the diabetic hearts from systolic impairment. Combined treatment with AT_1_ and AT_2_ blockers reduced protection provided by the AT_1_ blocker alone.

### Additional parameters of cardiac structure and function

Several other indices of cardiac structure and function, as measured by echocardiography, are presented in Table 1. There was no statistical difference in the heart rate among groups at the time of echocardiography. Cardiac output and stroke volume were reduced in diabetic mice compared to controls, which were significantly improved in all single treatment groups. The isovolumic contraction time was increased in the diabetic group, indicative of systolic dysfunction, as described above. Diastolic dysfunction was further confirmed by a decrease in the mitral annulus tissue Doppler velocities (E’/A’) in diabetic animals, which was prevented in the treatment groups. Significantly, left ventricular posterior wall thickness, the interventricular septum thickness (IVS), and the left ventricular internal dimension, measured in diastole, did not change over the course of diabetes, indicating no change in cardiac structure. However, the IVS measured in systole decreased, the significance of which is not known. The latter was prevented by all three RAS inhibitors.

**Table 1:** Echocardiography measurements in control, diabetic (10 wks), and treatment groups

	**Control**	**STZ + Veh**	**STZ + Alsk**	**STZ + Benz**	**STZ + Vals**	**STZ + PD + Vals**
HR	477 ± 20	473 ± 12	456 ± 21	452 ± 21	438 ± 13	447 ± 26
CO	24 ± 1.90	15.8 ± 0.62*	22.5 ± 1.66^†^	21.82 ± 1.98^†^	21.48 ± 1.87^†^	17.96 ± 0.84*
SV	52.8 ± 1.60	35.9 ± 1.60*	45.3 ± 2.40*^†^	47.51 ± 3.10^†^	47.82 ± 3.20^†^	39.76 ± 1.70*
IVCT	15.5 ± 0.60	17.9 ± 0.60*	15.2 ± 0.50^†^	15.2 ± 0.20^†^	15.5 ± 0.70^†^	16.8 ± 1.40
LVPWd	0.93 ± 0.03	0.90 ± 0.02	0.89 ± 0.03	0.94 ± 0.02	0.96 ± 0.04	1.15 ± 0.08^†^
LVPWs	1.29 ± 0.07	1.15 ± 0.09	1.37 ± 0.02	1.34 ± 0.06	1.28 ± 0.03	1.27 ± 0.06
IVSd	0.93 ± 0.03	0.87 ± 0.03	0.89 ± 0.03	0.98 ± 0.02^†^	0.93 ± 0.03	0.81 ± 0.04
IVSs	1.36 ± 0.05	0.96 ± 0.05*	1.38 ± 0.05^†^	1.40 ± 0.03^†^	1.30 ± 0.05^†^	1.21 ± 0.06^†^
LVIDd	3.91 ± 0.09	3.79 ± 0.10	3.42 ± 0.09*	3.47 ± 0.14*	3.68 ± 0.12	3.72 ± 0.12
LVIDs	2.83 ± 0.15	2.57 ± 0.13	2.34 ± 0.10*	2.42 ± 0.18	2.61 ± 0.10	2.67 ± 0.13
E'/A'	0.90 ± 0.04	0.67 ± 0.06*	0.84 ± 0.04	0.80 ± 0.05	0.81 ± 0.02	0.76 ± 0.11

For control group, n = 8; STZ + Veh, n = 9; STZ + Alsk, n = 8; STZ + Benz, n = 9; STZ + Vals, n = 7; STZ + Vals + PD, n = 7. CO = cardiac output (mls/min), SV = stroke volume (μl), IVCT = isovolumic contraction time (ms), LVPWd = left ventricular posterior wall thickness in diastole (mm), LVPWs = left ventricular posterior wall thickness in systole, IVSd = interventricular septum thickness in diastole (mm), IVSs = interventricular septum thickness in systole, LVIDd = left ventricular internal dimension in diastole, LVIDs = left ventricular internal dimension in systole (mm), E’A’ = mitral annulus tissue Doppler velocities. * = p < 0.05 vs. Control; ^†^ = p < 0.05 vs. STZ + Veh.

### Blood glucose levels and mean arterial pressure

**Figure 5:**
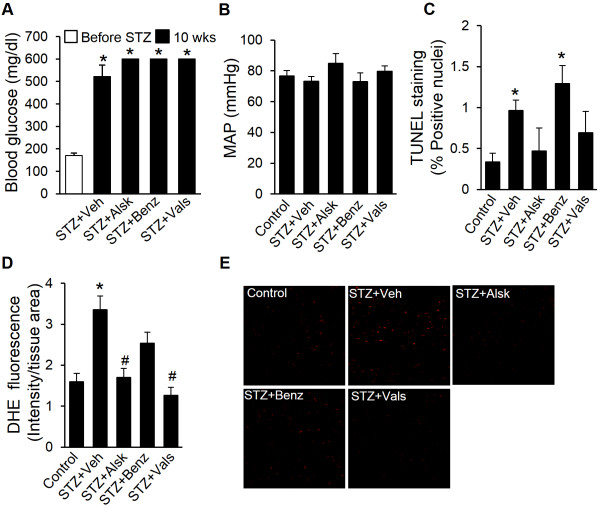
**Measurement of blood glucose, mean arterial pressure, oxidative stress, and cardiac myocyte apoptosis.** Blood glucose values shown are from animals before injecting STZ and after 10 wks of diabetes in different groups **(A)**. Mean arterial pressure (MAP) was calculated from blood pressure values measured through carotid artery cannulation following 10 wks of diabetes **(B)**. Apoptosis was measured by TUNEL staining of heart sections and is represented as TUNEL positive nuclei as a percentage of total nuclei **(C)**. Oxidative stress was measured by DHE staining **(D and E)**. The staining intensity was calculated from five images per heart, three hearts per group and normalized to the section area **(D)**. Representative images of DHE staining are shown **(E)**. Values are expressed as the mean ± SEM. *P < 0.05 vs. before STZ or Control, ^#^*P* < 0.05 vs. STZ + Veh.

Blood glucose levels were monitored biweekly and were consistently elevated. After 10 wks, diabetic animals exhibited elevated blood glucose levels (>500 mg/dl), which were not lowered by aliskiren, benazeprilat, and valsartan (Figure 5A). Mean arterial pressure (MAP) in AT_1a_-KO mice did not change as a result of hyperglycemia (Figure 5B), which is consistent with other reports in the STZ-induced model of diabetes [16]. Treatment with any of the RAS inhibitors did not reduce blood pressure, which corroborated previous reports of lack of hypotensive effects of these agents at comparable therapeutic doses in the absence of hypertension [17,18]. These observations suggested that the therapeutic effect of the RAS blockers on cardiac function were not secondary to the changes in hyperglycemia or blood pressure.

### Aliskiren and valsartan prevent diabetes-induced oxidative stress and apoptosis in the heart

Diabetic AT_1a_-KO mice showed a significant increase in DHE staining, compared to control mice. As shown in Figure 5D and E, the increased staining was completely abolished by aliskiren and valsartan; but, not by benazeprilat treatment. The number of apoptotic cells, detected by TUNEL staining, increased significantly in the diabetic heart (Figure 5C). The changes in apoptosis in diabetic and treated hearts were consistent with the changes in the oxidative stress. However, we did not observe an increase in cardiac fibrosis or a change in cardiac structure, as measured by LV posterior wall and interventricular septum thickness using echocardiography (data not shown). These results are consistent with our previous observations in WT mice [12].

### Cardiac AT_1b_ expression in AT_1a_-KO mice

**Figure 6:**
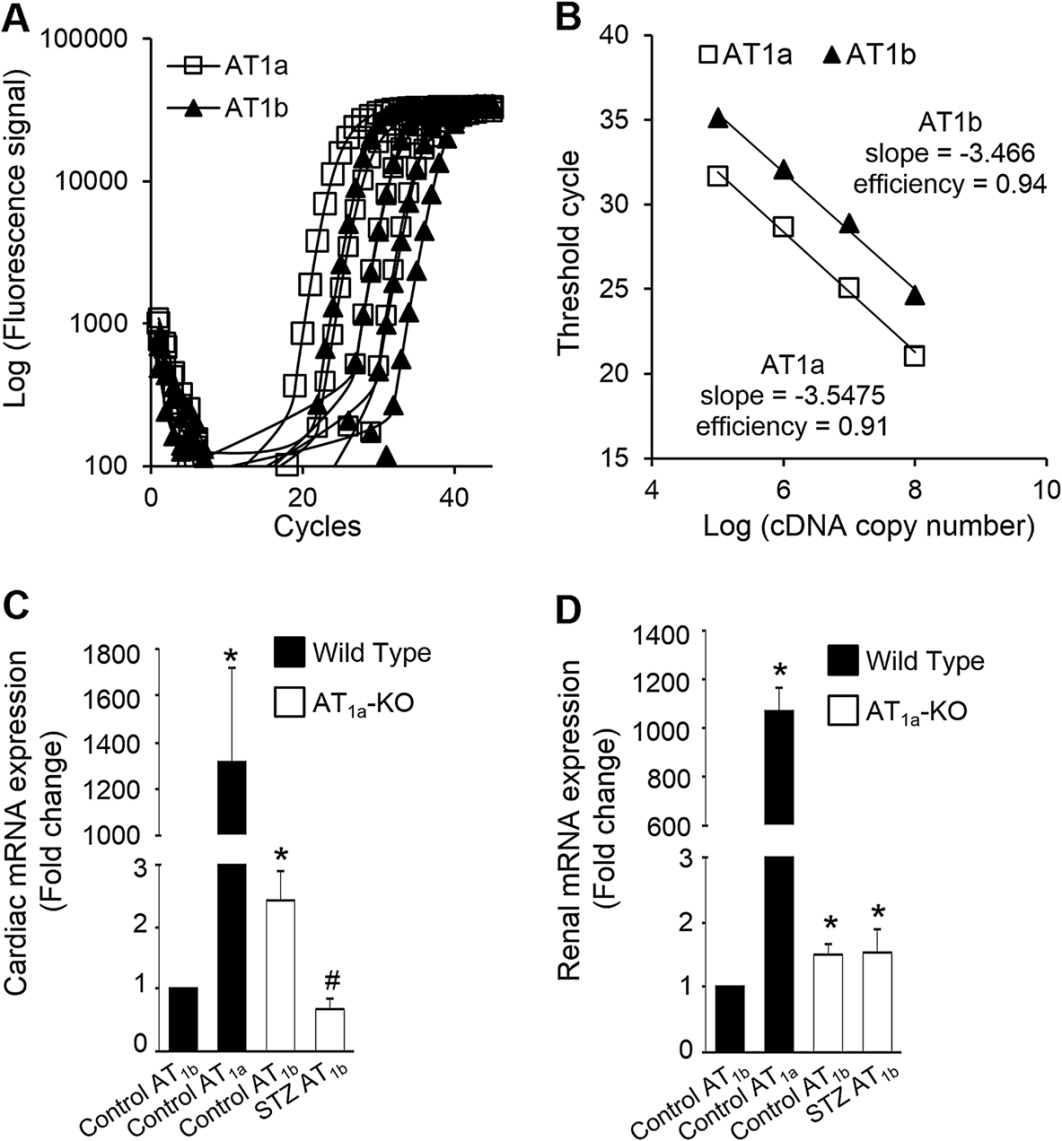
**Relative expression of AT**_**1a **_**and AT**_**1b **_**in WT and AT**_**1a**_**-KO mice.** Efficiency of the PCR for AT_1a_ and AT_1b_ was determined using serial dilutions of mouse adrenal gland cDNA **(A and B)**. Parallel lines of the amplification reactions demonstrate similar efficiency of both PCRs. Expression of AT_1a_ and AT_1b_ was measured by real-time RT-PCR in cardiac myocytes **(C)** and kidneys **(D)** of wild type and AT_1a_-KO (control and diabetic (STZ, 10 wk)) mice. Values are expressed as the fold change compared to control ± SEM. *P < 0.05 vs. control.

To determine any potential functional compensation by AT_1b_ in AT_1a_-KO mice, we measured mRNA levels of AT_1b_ in cardiac myocytes from WT mice, which were barely detectable with Ct values around 40. To compare the expression of AT_1a_ and AT_1b_, we determined the efficiency of the two PCRs, which were 0.91 and 0.94, respectively (Figure 6A and B). Comparatively, AT_1b_ expression was ~1/1316th of AT_1a_. In AT_1a_-KO mice, AT_1b_ was upregulated slightly but was still ~1/543th of AT_1a_ in WT mice (Figure 6C). Importantly, AT_1b_ expression was reduced in diabetic AT_1a_-KO mice, compared to non-diabetics. Renal mRNA levels of AT_1b_ and AT_1a_ were about 3-fold higher than the hearts; however, AT_1b_ expression remained scarce in comparison to AT_1a_ in WT mice. There was a small increase in renal AT_1b_ expression in AT_1a_-KO mice, but it was ~700 fold lower than renal AT_1a_ in WT mice (Figure 6D).

### Plasma renin levels are not changed following treatment with RAS inhibitors

**Figure 7:**
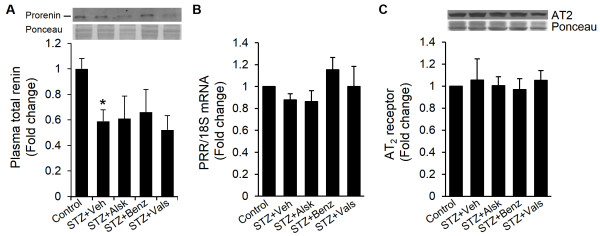
**Plasma renin levels and prorenin receptor and AT**_**2 **_**expression in cardiac myocytes.** Plasma renin **(A)** and AT_2_**(C)** levels were determined by Western blot analysis and PRR **(B)** expression was measured by real-time RT-PCR, in control, diabetic (STZ + Veh), and diabetic mice treated with different RAS inhibitors, after 10 wks of diabetes **(C)**. Representative Western blot images are shown. Values are expressed as the fold change compared to control ± SEM. *P < 0.05 vs. control.

RAS inhibitors cause a reactive rise in plasma renin by interfering with the negative feedback effects of ANG II on kidneys via AT_1_ receptor [19]. Similarly, we had previously reported an increase in plasma renin following RAS inhibition in WT mice [12]. To examine whether levels of AT_1b_ in AT_1a_-KO mice, though extremely low, are sufficient to cause a physiological response; we performed Western blot analysis of plasma samples, using an antibody which recognizes both prorenin and renin. As shown in Figure 7A, only prorenin was detected, which was reduced in diabetic animals compared to controls. None of the RAS inhibitors used in this study upregulated plasma prorenin. These results indicated that levels of renal AT_1b_ were not sufficient to respond to RAS inhibition in AT_1a_-KO mice; and by derivation, cardiac AT_1b_ receptor were also functionally irrelevant.

### Prorenin receptor and AT_2_ expression does not change in AT_1a_-KO mice following hyperglycemia or treatment with RAS inhibitors

Upregulation of the PRR in kidneys and hearts of diabetic animals has been reported, which may contribute to the pathological mechanism of tissue damage [20,21]. We determined mRNA and protein levels of the PRR in cardiac myocytes isolated from control, diabetic, and diabetic animals treated with RAS inhibitors. No significant change in PRR expression was observed in any of these groups, suggesting that the PRR may not have a role in diabetic cardiomyopathy in AT_1a_-KO mice (Figure 7B, protein data not shown). We also observed that AT_2_ receptor expression did not change in diabetes or following treatment with RAS inhibitors (Figure 7C).

### Aliskiren, benazeprilat, and valsartan normalize the kallikrein-kinin system in diabetic hearts

**Figure 8:**
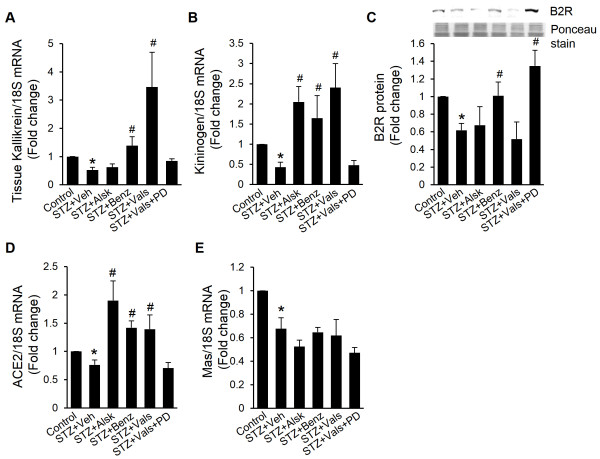
**Expression of tissue kallikrein, kininogen, kinin B2 receptor, ACE2, and Mas.** mRNA expression of tissue kallikrein **(A)**, kininogen **(B)**, ACE2 **(D)**, and Mas **(E)** in cardiac myocytes of control, STZ + Veh, STZ + Alsk, STZ + Benz, STZ + Vals, and STZ + Vals + PD after 10 wks of diabetes was measured by real-time RT-PCR and normalized to 18S mRNA. B2 receptor expression was measured by Western blot analysis **(C)**. A representative Western blot image is shown. Values are expressed as the fold change compared to control ± SEM. *P < 0.05 vs. control, ^#^*P* < 0.05 vs. STZ + Veh.

The kallikrein-kinin system (KKS) has been implicated in the development of diabetic cardiomyopathy [22,23]; and interaction between the KKS and the RAS at the level of ACE is well established [24]. In the present study, mRNA levels of tissue kallikrein and kininogen were reduced in isolated cardiac myocytes from diabetic hearts. Treatment with benazeprilat and valsartan reversed the effect of diabetes on these two genes, whereas aliskiren treatment only normalized kininogen mRNA expression (Figure 8A and B). Protein expression data is not presented, as available antibodies did not recognize tissue isoforms of mouse kallikrein and kininogen with certainty. Further, the protein expression of B2 receptor, which is responsible for the cardioprotective actions of bradykinin, was significantly diminished in diabetic hearts (Figure 8C). Benazeprilat significantly normalized the protein expression of B2 receptor, whereas aliskiren and valsartan did not have any effect. Together, these results suggest that part of the beneficial effects of RAS inhibitors was mediated by the KKS.

### Effect on ACE2/Ang 1-7/Mas pathway

ACE2/Ang 1-7/Mas pathway represents a protective mechanism in the heart, which is downregulated in diabetes (Figure 8D and E). We observed that the expression of ACE2 was enhanced in all treatment groups except in the STZ + Vals + PD group. A reduction in Mas expression in diabetes was not reversed by any treatment.

## Discussion

In this study, we determined a role of the intracellular cardiac RAS in the development of diabetes-induced cardiac dysfunction. We report three significant findings: 1) activation of the intracellular RAS, indicated by increased intracellular levels of ANG II in the absence of AT_1a_-mediated uptake; 2) development of cardiac dysfunction in AT_1a_-KO mice, which is prevented by a renin inhibitor and; 3) prevention of cardiac dysfunction by an ACE inhibitor and ARB in AT_1a_-KO mice, possibly through activation of the kallikrein-kinin system.

The basic premise of this study was the activation of the intracellular cardiac RAS in diabetes. In our previous studies, demonstration of intracellular ANG II synthesis was based on an increase in cellular ANG II levels, after pharmacological blockade of ANG II uptake by cells using an ARB [8,9]. Here we demonstrated activation of the intracellular RAS in a genetic model of AT_1a_-deficiency. We measured AGT and renin expression and ANG II concentration in cardiac myocytes isolated from AT_1a_-KO mice after one wk of diabetes. A significant upregulation of AGT and renin expression was observed, which was accompanied by a several fold increase in ANG II levels in AT_1a_-KO mice. The increase in ANG II levels was similar to that in WT mice (Figure 2). Intracellular ANG II synthesis in cardiac myocytes of AT_1a_-KO mice was likely due to retention of AGT and renin inside cells in response to high ambient glucose levels, as we had reported in neonatal rat ventricular myocytes [9]. However, the intracellular site of synthesis and mechanism of renin activation in cardiac myocytes are not known. Since intracellular ANG II in AT_1a_-KO mice could not have been due to cellular uptake, these observations clearly demonstrated activation of the intracellular RAS in the diabetic heart.

Rodents express two subtypes of AT_1_ receptor, AT_1a_ and AT_1b_. It may be argued that some of the observations in the current study could be related to AT_1b_ receptor. Several studies have examined tissue-specific expression of AT_1a_ and AT_1b_ in mice and rats. In rats, one study reported solely AT_1a_ expression in the heart, another study detected very low AT_1b_ expression, about 1/10th of AT_1a_ expression [25,26]. However, in mice, AT_1b_ expression was detected only in the adrenal gland, brain, and testis; not in the heart [27]. In another mouse study, in which the AT_1b_ coding exon was replaced with the reporter gene lacZ, there was no detectable lacZ expression in kidneys and heart [28]. Consistent with these studies, we observed extremely low levels of cardiac myocyte AT_1b_ expression, i.e., 1/1316th and 1/543th in WT and AT_1a_-KO mice, respectively, as compared to AT_1a_ expression in WT mice. AT_1b_ expression was detectable because we performed PCR up to 40 cycles, while routine protocols use only 30 cycles. Kidneys had higher expression of AT_1b_ compared to the heart; however, were not responsive to RAS inhibitors, as shown by the lack of a reactive rise in renin expression. These observations suggested that the levels of AT_1b_ in the heart were not sufficient to be physiologically relevant. Functional studies in AT_1a_-KO or AT_1a_-AT_1b_ double knockout mice by other investigators also did not find any role for AT_1b_ in the heart [29,30]. Further, AT_1b_ expression was reduced in the diabetic group compared to controls, indicating that AT_1b_ did not contribute to diabetes-induced cardiac dysfunction. To determine the role of the AT_2_ receptor, we treated diabetic animals with the AT_2_ antagonist PD123319, along with valsartan. Treatment with the AT_2_ blocker reduced the protective effects of valsartan, suggesting that the AT_2_ receptor had a beneficial role and was not involved in diabetes-induced cardiac dysfunction.

Nuclear AT_1_ and AT_2_ receptors have been described in cardiac myocytes, which respond to ANG II stimulation by initiating nuclear signaling events [31]. Genetic removal of AT_1a_ and the lack of functional levels of AT_1b_ expression would indicate that AT_1a_-KO mice will not have cardiac nuclear AT_1_ receptors. AT_2_ has a protective role, as discussed above. Thus, a role of the known nuclear ANG II receptors in intracellular ANG II-mediated diabetic cardiomyopathy is unlikely. Further studies are required to understand the mechanism of intracellular ANG II-induced diabetic cardiomyopathy.

Given that RAS inhibitors are cardioprotective in diabetes, the development of cardiac dysfunction in diabetic AT_1a_-KO mice represents an important finding. A decrease in systolic and diastolic function and increase in oxidative stress and apoptosis in the heart of diabetic animals was observed. The systolic and diastolic dysfunction in AT_1a_-KO mice appeared similar to that we described in WT animals [12]. The onset of cardiac dysfunction was slightly delayed (E/A: 6 wks vs. 8 wks in WT vs. AT_1a_-KO mice, respectively) and the degree of severity was less after 10 wks of diabetes in AT_1a_-KO mice (E/A: 1.11 vs. 1.24; IVRT: 19.8 vs. 18.7 ms; EF: 47 vs. 52%; FS: 23 vs. 26%; in WT vs. AT_1a_-KO mice, respectively). However, these differences could be due to inter-experimental variation. These observations suggested that the development of diabetic cardiomyopathy was independent of AT_1a_. A similar observation was made regarding STZ-induced diabetic nephropathy in AT_1a_-KO mice [20]. It was reported that the AT_1a_ deficiency delayed the onset of diabetes-induced glomerulosclerosis and proteinuria; but, did not prevent full development of these pathological endpoints [20]. Treatment with the ACE inhibitor imidapril partially prevented diabetic nephropathy; these investigators did not study the effect of an ARB. However, a handle region peptide, that blocked binding of prorenin to the prorenin receptor (PRR) and inhibited downstream signaling, completely prevented glomerulosclerosis and proteinuria. Based on these observations, the investigators concluded that an ANG II-independent mechanism contributed to the development of diabetic nephropathy [20]. We observed no change in PRR expression in cardiac myocytes of diabetic AT_1a_-KO animals or following treatment with RAS inhibitors, compared to non-diabetic controls, suggesting that the PRR was not involved in cardiac dysfunction in AT_1a_-KO mice. We had previously observed increased cardiac PRR in diabetes in WT mice [12]. Given that PRR expression is increased in conditions that activate the RAS (low salt and hyperglycemia) and is decreased by RAS inhibition, we believe that AT_1_ receptor deficiency likely explains the lack of PRR upregulation in this study. Further, development of diabetic cardiomyopathy in AT_1a_-KO mice supported our hypothesis and previous reports of AT_1_-independent actions of intracellular ANG II in the heart [8,15,32]. However, to confirm the involvement of ANG II, we studied whether blocking ANG II synthesis with a renin inhibitor would prevent diabetes-induced cardiac dysfunction in AT_1a_-KO mice. We also investigated whether an ACE inhibitor or ARB would provide protection via non-RAS mechanisms, as was suggested by our previous study in WT mice [12].

We observed that the renin inhibitor aliskiren completely protected animals from developing cardiac dysfunction up to 10 wks of diabetes. We have previously reported that high glucose-induced intracellular ANG II synthesis is both renin and chymase-dependent in cardiac myocytes and renin and ACE-dependent in cardiac fibroblasts [9,33]. Aliskiren accumulates in tissues, including cardiac myocytes and therefore inhibits renin-dependent intracellular ANG II production [8,34,35]. We previously reported that aliskiren was better than benazeprilat and candesartan in reducing cardiac ANG II levels and preventing diabetes-induced cardiac myocyte apoptosis, oxidative stress, and cardiac fibrosis after one wk of diabetes in rats [8]. In the current study, preservation of heart function by aliskiren in AT_1a_-KO animals, suggested the involvement of intracellular ANG II in diabetic cardiomyopathy.

Significantly, benazeprilat and valsartan also showed protective effects, similar to aliskiren, in AT_1a_-KO mice. Regarding protection by the ACE inhibitor, it may be argued that ACE-mediated ANG II synthesis in the circulation or by cardiac fibroblasts had a role in cardiac dysfunction. However, in the absence of AT_1a_ receptor, extracellular ANG II would be ineffective in the heart. The latter argument also applies to any change in the circulating RAS, as a result of AT_1a_ deficiency or the RAS inhibitor treatment. It has been reported that ACE inhibitors also have cardioprotective effects through inhibition of kinin degradation [24,36]. Additionally, these drugs enhance kinin B1 and B2 receptor function via allosteric mechanisms that involve ACE and B2 receptor heterodimerization and direct binding of ACE inhibitors to B1 receptor [37]. Beneficial effects of kallikrein overexpression and B2 receptor signaling, and detrimental effects of B1 receptor-induced inflammation, have been described in diabetic cardiomyopathy [23,38,39]. In this study, benazeprilat normalized the expression of tissue kallikrein, kininogen, and B2 receptor, which were impaired in diabetic animals, suggesting a role of the KKS in cardioprotection by benazeprilat in AT_1a_-KO mice. The blockade of the B2 receptor reduced the renal protective effects of the ACE inhibitor ramipril in db/db mice, further supporting our conclusion [40].

Particularly intriguing was the observation that the AT_1_ receptor blocker valsartan was as effective as the other two drugs, despite the lack of AT_1a_ receptor in these animals. As discussed above, the mouse heart does not express physiologically relevant levels of AT_1b_; thus, the protective effects of valsartan were likely mediated via non-RAS mechanisms. Valsartan was recently shown to markedly inhibit lipopolysaccharide-induced cytokine production in macrophages and improve insulin resistance in co-cultured adipocytes, via an unidentified AT_1a_ receptor-independent pathway [41]. Another ARB, losartan, protected WT mice against myocardial ischemia-reperfusion injury, which was not observed in kallikrein gene-deficient mice, suggesting that the KKS was a major determinant of the cardioprotective effects of losartan [42]. Similarly, the inhibitory effects of valsartan on ANG II-induced MAP and urinary sodium excretion in healthy humans were shown to be mediated via the B2 receptor [43]. We observed that valsartan significantly increased mRNA expression of tissue kallikrein and kininogen, which might have contributed to the effects of valsartan in this study. To determine whether these actions of valsartan on the KKS were mediated through AT_2_ receptor, as suggested by some studies [42,44], we measured AT_2_ expression and treated diabetic mice with the AT_2_ antagonist PD123319 in combination with valsartan. No change in AT_2_ expression was observed following diabetes or treatment with valsartan; however, we did observe reduced protection by valsartan when used in combination with PD123319. Further, activation of the KKS, with the exception of B2 receptor, was blunted with the co-treatment, suggesting a contribution of AT_2_ in cardiac protection. It is noteworthy that functional AT_2_ receptors on cardiac myocyte nuclei have been demonstrated, which are accessible to intracellular ANG II [31]. However, how valsartan would modulate AT_2_ function in AT_1a_-KO mice is not clear. Aliskiren treatment also increased kininogen levels; thus, a partial contribution of the KKS in aliskiren-mediated cardioprotection cannot be excluded [45]. ACE2 expression was increased in animals treated with all three RAS inhibitors, suggesting increased Ang 1–7 generation; however, Mas expression was not rescued by these treatments. Therefore, a role of the Ang 1-7/Mas axis, independently or in concert with the KKS, in cardioprotection is not clear.

## Conclusions

In conclusion, we demonstrated an elevation of cardiac intracellular ANG II and the development of diabetes-induced cardiac dysfunction in AT_1a_-KO mice. All three classes of RAS inhibitors protected the heart against diabetic cardiomyopathy. Whereas protection provided by a renin inhibitor suggested the involvement of intracellular ANG II in the pathological process, the beneficial effects of the ACE inhibitor and ARB confounded the interpretation. Two possibilities emerged from this study; either the RAS did not have a significant role in the development of diabetic cardiomyopathy, or if it did, then it was likely via the intracellular RAS. Interestingly, our knowledge of the role of the RAS in diabetes-induced organ damage is based entirely on pharmacological benefits of RAS inhibitors. More studies utilizing genetic animal models are required to determine precisely the role of ANG II in diabetic tissue damage. If a role of intracellular ANG II in diabetic cardiomyopathy is established, delineation of AT_1_-independent mechanisms of ANG II would require substantial future research. In summary, this study provides new insights into the role of the RAS and RAS inhibitors in diabetic cardiomyopathy.

### Limitations

This study focused on the role of cardiac intracellular RAS in diabetic cardiomyopathy. Thus, isolated cardiac myocytes, rather than whole hearts, were used to measure various molecular parameters, which severely limited the amount of sample available for analysis. For the latter reason, ANG II levels were not measured in the treated groups. However, we had reported in wild type mice and rats that AT_1_ receptor blockers and ACE inhibitors did not significantly reduce intracellular ANG II levels in cardiac myocytes from diabetic hearts. Only a renin inhibitor prevented diabetes-induced ANG II formation [8,12]. We did not expect different results in AT_1a_-KO animals. Any change in the circulating ANG II levels, as a result of the treatments with RAS inhibitors, would not be of any consequence in the heart of AT_1a_-KO animals. Another limitation of this study is that gene expression data for some parameters described in Figure 8 could not be confirmed by protein analysis due to non-specific bands on Western blots that precluded identification of protein-specific bands with certainty. Regarding the mechanism of action of benazeprilat and valsartan via the KKS, our data are consistent with the literature; however, more studies will be required. Finally, whereas our hypothesis of the role of intracellular ANG II in diabetic cardiomyopathy is strengthened by the data presented, extended studies will be required to unequivocally prove it.

## Abbreviations

ACE: Angiotensin-converting enzyme; ACE2: Angiotensin-converting enzyme; ACEi: ACE inhibitor; AGT: Angiotensinogen; Alsk: Aliskiren; Ang-(1–7): Angiotensin-(1–7); ANG I: Angiotensin I; ANG II: Angiotensin II; ARB: Angiotensin receptor blocker; ANP: Atrial natriuretic peptide; AT1 receptor: AngII type 1 receptor; AT1a-KO: AT1a receptor-deficient; AT2 receptor: AngII type 2 receptor; B2R: B2 receptor; Benz: Benazeprilat; BNP: Brain natriuretic peptide; CO: Cardiac output; DHE: Dihydroethidium; E/A ratio: Ratio of mitral valve flow velocities; E’/A’ ratio: Ratio of early to late mitral annulus tissue Doppler velocities; ERK: Extracellular-signal-regulated kinase; EF: Ejection fraction; FS: Fractional shortening; IVCT: Isovolumic contraction time; IVRT: Isovolumic relaxation time; IVSd: Interventricular septum thickness at end-diastole; IVSs: Interventricular septum thickness at end-systole; KKS: Kallikrein-kinin system; KLK: Kallikrein; KNG: Kininogen; LV: Left ventricular; LVIDd: LV internal dimension at end-diastole; LVIDs: LV internal dimension at end-systole; LVPWd: LV posterior wall thickness at end-diastole; LVPWs: LV posterior wall thickness at end-systole; Mas: Mas receptor; MHC: Myosin heavy chain; MAP: Mean arterial pressure; PRR: (Pro)renin receptor; RAS: Renin–angiotensin system; ROS: Reactive oxygen species; SV: Stroke volume; TUNEL: Terminal deoxynucleotide transferase-mediated dUTP nick-end labeling; Vals: Valsartan; Veh: Vehicle; WT: Wild type.

## Competing interests

RK and KMB had previously received research funding from Novartis Pharmaceuticals Corporation. In addition, Novartis provided aliskiren, valsartan, and benazeprilat for use in this study. CMT, QCY, RS and NC do not have any conflicts of interest.

This work was published in the abstract form in *Hypertension* 58(5):e128, 2011.

## Authors’ contributions

QCY researched data and wrote the manuscript, CMT researched data and reviewed the manuscript, RS researched data, NC researched data, KMB reviewed/edited the manuscript and contributed to discussion, RK designed and supervised research and wrote the manuscript. KMB and RK are guarantors of this work and, as such, had full access to all the data in the study and take responsibility for the integrity of the data and the accuracy of the data analysis. All authors read and approved the final manuscript.
